# Biotechnological Innovations from Ocean: Transpiring Role of Marine Drugs in Management of Chronic Disorders

**DOI:** 10.3390/molecules27051539

**Published:** 2022-02-24

**Authors:** Saurabh Bhatia, Rashita Makkar, Tapan Behl, Aayush Sehgal, Sukhbir Singh, Mahesh Rachamalla, Vasudevan Mani, Muhammad Shahid Iqbal, Simona Gabriela Bungau

**Affiliations:** 1 Natural and Medical Sciences Research Center, University of Nizwa, Birkat Al Mauz 616, Nizwa P.O. Box 33, Oman; sbsaurabhbhatia@gmail.com; 2 School of Health Science, University of Petroleum and Energy Studies, Dehradun 248007, India; 3 Chitkara College of Pharmacy, Chitkara University, Patiala 140401, India; rashitamakker32@gmail.com (R.M.); aayushsehgal00@gmail.com (A.S.); sukhbir.singh@chitkara.edu.in (S.S.); 4 Department of Biology, University of Saskatchewan, 112 Science Place, Saskatoon, SK S7N 5E2, Canada; maheshgupta65@gmail.com; 5 Department of Pharmacology and Toxicology, College of Pharmacy, Qassim University, Buraydah 51452, Saudi Arabia; vasumpharmacol@gmail.com; 6 Department of Clinical Pharmacy, College of Pharmacy, Prince Sattam bin Abdulaziz University, Alkharj 11942, Saudi Arabia; m.javed@psau.edu.sa; 7 Department of Pharmacy, Faculty of Medicine and Pharmacy, University of Oradea, 410073 Oradea, Romania; 8 Doctoral School of Biomedical Sciences, University of Oradea, 410087 Oradea, Romania

**Keywords:** marine drugs, diabetes mellitus, cancer, cardiovascular disorders, neurodegeneration

## Abstract

Marine drugs are abundant in number, comprise of a diverse range of structures with corresponding mechanisms of action, and hold promise for the discovery of new and better treatment approaches for the management of several chronic diseases. There are huge reserves of natural marine biological compounds, as 70 percent of the Earth is covered with oceans, indicating a diversity of chemical entities on the planet. The marine ecosystems are a rich source of bioactive products and have been explored for lead drug molecules that have proven to be novel therapeutic targets. Over the last 70 years, many structurally diverse drug products and their secondary metabolites have been isolated from marine sources. The drugs obtained from marine sources have displayed an exceptional potential in the management of a wide array of diseases, ranging from acute to chronic conditions. A beneficial role of marine drugs in human health has been recently proposed. The current review highlights various marine drugs and their compounds and role in the management of chronic diseases such as cancer, diabetes, neurodegenerative diseases, and cardiovascular disorders, which has led to the development of new drug treatment approaches.

## 1. Introduction

Drug molecules derived from marines sources are highly heterogenous in nature due to the abundant coverage of oceans, which thereby host the lives of a wide diversity of species [1,2]. In a pharmacological preclinical study, about 75 natural compounds that were isolated from marine organisms significantly showed biological and therapeutic activities [3,4]. The first marine drug from the cone snail peptide—namely, ziconotide, under the trade name Prialt—was approved in 2004 in the United States for the management of spinal cord injury, mediating chronic pain. Later, in 2007, another marine product named trabectedin, a sea squirt metabolite, was also approved by the European Union for the treatment of soft tissue sarcoma [5]. The drugs obtained from marine sources have displayed an exceptional potential in the management of various types of chronic diseases, including cancer, due to their potent anticancer activities [5,6,7]. These drugs have also gained the great interest of those developing new antimicrobial agents. Sponges belonging to the phylum *Porifera*, are the oldest and most prolific marine organisms on the planet. Demospongiae, a class of *Porifera*, account for 83% of the species with the highest number of bioactive compounds [8]. Another marine genus named *Lendenfeldia* is rich in sulfated steroids, and its metabolites possess anti-HIV, anti-inflammatory, antitumor, and antifouling activities. Drug discovery programs have significantly increased their exploration of lead molecules from marine natural products, and a higher number of bioactive products have been screened for their activity and are under development in clinical trials [9]. Terrestrial plants such as digitalis, morphine, and many other natural compounds have served as drug molecule since olden times. However, the modern pharmaceutical industry has expanded a keen interest in developing drug molecules from marine sources, as they are believed to provide more novel and potent drug compounds since they can survive under extreme conditions, such as the photodynamic and extreme temperatures, pressure, and oxidative stress of the ocean [10]. The basic scientific research in pharmacology and chemistry of marine-derived natural products mainly began in the early 1970s and has now finally begun to bear fruit due to advances in analytical and screening techniques that have fastened the drug discovery process [11]. The current review highlights the drugs obtained from marine natural products and their role in the treatment and management of chronic diseases such as cardiovascular and neurodegenerative diseases, diabetes mellitus, and cancer. These drugs offer a look into the future of promising products that can be obtained from the sea.

The development of drugs from marine sources is a highly tedious process, as it is difficult to procure and manufacture quantities of novel drug leads from marine sources [12]. For instance, the marine sponges are highly chemically versatile in nature and act as a resource of developmental compounds, such as hemiasterlin and discodermolide, which are extracted from primitive metals found in marine habitats. Sponges are extremely treasured since they are difficult to extract, and specimens are mostly collected by hand during deep and shallow water scuba diving, but also with the help of submarines equipped with robotic arms. These techniques are highly expensive and result in an uncertain yield, thereby posing a great challenge to those developing medicines and the pharmaceutical industry. Nonetheless, the interest of researchers in marine products has remained intact, which has led to the budding of innovative solutions to overcome the challenges [13,14]. The story of discovery of therapeutic marine compounds begins with the identification of a marine source and the further isolation of a therapeutic target by analytical techniques. Once a bioactive compound is isolated, it is further studied for its structure and is presented into preclinical and clinical studies for an estimation of its activity. The discovery of drugs from marine sources has also led to the development of genome mining techniques, which have a tendency to improve future discovery processes.

## 2. Identification and Isolation of Bioactive Compound from Marine Natural Extract

Natural products along with their structural analogues have contributed to pharmacotherapy throughout history, especially in the management of infectious and cancerous diseases [15,16]. However, the major challenge associated with natural products has been drug discovery, due to poor techniques of screening, isolation, characterization of the drug, and its optimization—all of which has now been overcome by advances in analytical tools such as gene mining and advanced techniques of microbial culturing, which has revitalized the interest of the pharmaceutical industry in identifying drug leads from marine sources, opening several new treatment opportunities [17,18]. The natural drugs are typically products with higher molecular mass [19,20], and they present several advantages which are discussed in this article, yet they have several drawbacks, which have led pharmaceutical companies to reduce their efforts in the discovery of natural product-derived molecular leads [21]. The screening of natural products typically consists of an extract library mainly derived from the natural sources that may not necessarily be compatible with traditional target-based assays, making it tedious to identify bioactive compounds of interest [22]. Several tools and techniques are applied to assess whether a new molecule has been discovered or whether it is merely a rediscovery of already known compounds—a process that can be very challenging [23]. In addition, a major hurdle faced by the pharmaceutical industry is obtaining intellectual property rights for an unmodified natural product that has relevant bioactivity, as the naturally occurring compounds cannot always be patented in their native forms; however, the simpler molecules with biological activity can be patented easily [24]. The complex structures of natural products are advantageous in generating structural analogues for exploring the structure’s activity relationships and optimizing them for targeted mechanism of action [25]. The modern techniques—which include genome mining, genome engineering, and advances in analytical procedures and systems of cultivation—have led to an increased emphasis on drug development from marine sources, as they have helped overcome many of the major challenges that were being faced by the researchers—techniques that have proven to be promising [26,27]. 

**Figure 1:**
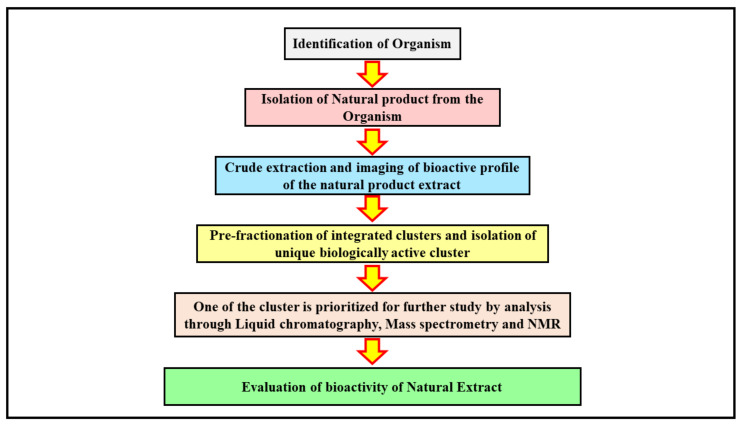
The figure illustrates the process of isolation of a bioactive molecule from a marine source. The identification of a specific organism is a first step in determining the bioactive lead moiety. Once the marine source with a desired therapeutic effect is discovered, the natural products within it are obtained by extraction processes. The crude extract collected at the end is further studied using imaging techniques to understand its bioactive profile. The integrated clusters of the bioactive compound in the crude extract solution are further pre-fractionated to obtain unique clusters that are biologically active. The active clusters are analyzed through advanced analytical techniques such as NMR, mass spectroscopy, and liquid chromatography for their structure elucidation and bioactivity determination, and the desired cluster is carried forward for further studies. The bioactive lead molecule selected is evaluated for its therapeutic activity through pre-clinical and further clinical trial studies.

The application of advanced analytical techniques begins with the screening of crude drug extracts, followed by further isolation and identification of a bioactive molecule that is fractionated to obtain the active moiety from the natural product [28]. The isolation of a bioactive compound is a laborious task and is highly challenging. The molecule isolated is run through the extract libraries and further exposed to high throughput screening so that the crude extract can be pre-fractionated into subfractions that are more suitable for the system that handles automated liquids [3]. The methods of fractionation can be altered to obtain preferential subfractions with active compound drugs that are alike in nature. This process can increase the number of hits compared with the compounds obtained from crude extracts, which enables more efficient and promising hits [6]. An advance in the instrumentation used in analytical methods, combined with advanced computational approaches, can lead to isolation of possible analogue structures of natural products [29]. The precise information about the metabolic composition of the crude marine extract can be obtained through metabolomics, which helps in prioritizing the isolation of a compound and its dereplication to annotate the structural analogues of the newly derived product [30] (Figure 1).

The current review highlights the role of marine drugs in the management of cancer, diabetes mellitus, cardiovascular disorders, and neurodegenerative diseases which are listed below.

## 3. Marine Drugs in the Management of Cancer

Cancer is a chronic disease and can be found in almost all multicellular organisms. The disease is strongly associated with aging because, with increasing age, mutations within the somatic cells accumulate and promote unregulated growth and invasion of dysfunctional cells, which leads to altered functions in the body and works against the organism’s health. Apart from the uncontrolled cellular growth, this disease also displays genetic instability, evasion of growth suppressors, immortality of replication, resistance to cell death, angiogenesis initiation, energy metabolism reprogramming, prevention of immune destruction, tumor-derived inflammation, and metastasis [31,32,33]. Advanced research and technology combined with efforts to determine and understand the hallmarks of cancer have led to improvement in the clinical outcomes of cancer patients by developing newer and more novel diagnostic techniques and therapeutic medications. The higher costs of these therapies and the sometimes extension of patient survival by just few months pose a major challenge to ongoing treatment, thereby provoking a dire need to identify promising new therapies for the management of this life threating disease. There are two general classes of cancer therapies: cancer therapies derived from natural compounds and cancer therapies derived from synthetic compounds—each further subcategorized into small molecules or low molecular weight substances that elicit biological responses by entering cells readily and biologics or large molecular weight substances such as ribonucleic acid (RNA) or monoclonal antibodies that are penetrated across cells with the help of delivery systems [34]. The majority of the cancer treatment drugs are naturally derived substances. For instance, the most primarily used chemotherapeutic drugs for the management of prostrate, breast, and other cancers—namely, docetaxel and paclitaxel—are derived from the taxanes plant. Cabazitaxel, another naturally derived anticancer compound was derived by chemical diversification of taxanes [35]. Factually, most of the anticancer drugs derived from natural resources are derived from terrestrial ecosystems, and about 100,000 compounds have been isolated from plants alone. About 99% of the total living space on earth is deep ocean, and oceans are where80% of the entire species in the world live. In the recent years, the interest of researchers has progressively focused on the marine environment, and researchers have successfully isolated over 2000 compounds over the past three decades [36]. The richness in species of the ocean and its extraordinary diversity with a large temperature and pressure tolerance window, presence of variety of chemicals and metals, saline nature, low to bright light, and allelopathic defenses has attracted the pharmaceutical industries towards the ocean, despite the small number of compounds isolated from it to date. Marine sources are believed to have treasurable therapeutic potential based on the unique dwelling inhabitant and hence the ocean is being explored for its hidden potential. Its noteworthy that the products derived from the ocean are extremely potent and act through multiple molecular pathways and collectively have an ability to target different hallmarks of cancer [37]. Some of the anticancer compounds isolated from marine sources are listed below.

Over 1000 compounds have been isolated from marine sources and are being tested for their activity in preclinical studies; 23 marine derived compounds are under clinical trials between phases I to III, and 7have been approved for marketing. Four compounds out of the total number of marine-derived molecules—namely, trabectedin, cytarabine, eribulin mesylate, and brentuximab vedotin, a conjugated antibody—are being used clinically for their anticancer properties [38].

### 3.1. Cytarabine (Cytosar)

Cytarabine is the debutant lead molecule that has been isolated from the ocean for the management of cancer. Cytarabine was developed by the synthesis of analogs of natural arabino nucleosides and cytosine arabinose from the Caribbean sponge *Cryptotethya crypta*. The chemical structure of this anticancer compound has been found to be related to Spong uridine and Spong thymidine, which are natural products isolated from the marine sponge *Tectitethya cripta*. Cytarabine acts by rapidly converting into its respective triphosphates arabinonucleoside by phosphorylation in a sequential manner. It is an antimetabolite drug with a structure that is sufficiently similar to natural metabolites of the body and acts by interfering with their functioning and hence preserving normal cellular metabolism [39]. The triphosphocytarabine formed upon phosphorylation becomes a substrate for DNA polymerase and subsequently is amalgamated in place of cytosine within the DNA. The arabinose is implanted instead of deoxyribose upon binding of DNA with cytarabine triphosphate and promotes elongation of the DNA strain by preventing phosphodiester bonding between the two pentose sugars, thereby prohibiting synthesis of DNA and hampering abnormal cellular growth. This drug was first approved for its clinical applications in 1969 and has been used in the management of wide array of leukemias, such as non-Hodgkin’s lymphoma, acute lymphocytic leukemia, chronic myelogenous leukemia, etc. Cytarabine has been claimed to be the foremost example of a commercially available marine-derived drug, even though the molecule itself is not a natural product but a structural analog [40]. The use of adenine arabinose analogs has also been noted in the development of vidarabine, an antiviral drug used in the management of varicella zoster and herpes simplex virus [41]. Furthermore, another antiviral drug, azidothymidine, has also reportedly been found to be extremely effective in the management of acquired immune deficiency syndrome (AIDS) by blocking the activity of the reverse transcriptase enzyme of the virus, which is highly crucial for replication of the human immune virus (HIV).

### 3.2. Trabectedin

Trabectedin was identified as one of the most abundant structurally related alkaloids that has been isolated from the Caribbean ascidian *Ecteinascidia turbinate*. The drug was first isolated with a very complex procedure in 1996. This molecule acts as an alkylator of DNA but differs from the usual alkylating agents. Trabectedin binds with the guanine residues of the double helix DNA and generates specific sequences, causing bending of strands in a direction opposite to the site of alkylation. These trabectedin abducts arrest the activity of RNA polymerase II and prevent the transcription of DNA, thereby preventing abnormal cellular growth. The most prominent effect of this drug is the inhibition of transcription of MDR1 genes, which are chiefly responsible for producing P glycoproteins and initiating the detoxification processes in the cells. The drug also successfully prevents the repair of DNA lesions due to the rest of RNA polymerase II, thereby producing a significant impact on the tumor microenvironment. Trabectedin also activates caspase 8 protein, which further induces apoptosis in macrophages and monocytes, thereby prohibiting the release of inflammatory mediators and the growth of angiogenic factors and preventing metastasis. Trabectedin acts via DNA alkylation by binding to the guanine residues, further generating exclusive sequences that cause bending of the double helix strands in a direction opposite to that of alkylation and differing greatly from other alkylating agents. The action of RNA polymerase II is arrested with simultaneous DNA transcription prohibition, hence preventing the abnormal growth of the cells. The drug prominently acts by inhibiting MDR1 genes transcription, which chiefly indulging in the synthesis of P glycoproteins, thereby stimulating the process of cellular detoxification [42]. The repair of the lesions in DNA is also counteracted by this drug due to RNA polymerase II arrest, hence producing a significant effect in the deterioration of the environment of the tumor.

### 3.3. Eribulin Mesylate

Eribulin is a mesylate salt prototype of high molecular weight halichondrin B, obtained from a class of halichondrins, a progression of macrocyclic polyethersthathave anticancer activity. The drug is obtained from *Halichondria okadai* sponges found on the coast of the Miura Peninsula in Japan. The macrocyclic ring of halichondrin B is responsible for the anticancer activity, as determined by the structure–activity relationship. This drug is believed to be the most complex drug synthesized. This naturally derived potent antimitotic drug mainly acts by inhibiting the microtubule. Tubulin is the ultimate target of this compound, and it prevents polymerization by binding to it and further arresting the microtubular extension. This irreversibly blocks the mitosis of the cells, and its prolongation finally induces apoptosis-mediated cell death. The drug eribulin mesylate was approved in 2010 by the FDA for the management of advanced metastatic breast cancer, and it was approved as second-line treatment for liposarcoma therapy in 2016 [43].

### 3.4. Brentuximab Vedotin

In 1972, anticancer activity was discovered from the extract of *Dolabella auriculria,* a gastropod mollusk found in the Indian Ocean. After 15 years of continuous research, the peptides—namely, dolastatins—were identified as being the chief active compounds that solely possess potent antiproliferative activity against tumor cells [43]. These peptides led to microtubule blockage and polymerization, consequently preventing rapid tumor cell division and hence prohibiting the growth and proliferation of tumor cells in the body. Brentuximab vedotin has an antibody drug conjugate structure and is mainly developed with a dolastatin10-derived natural product molecule, monomethylauristatin E, isolated from the mollusk *Dolabella auricularia*. In 2012, Adcetris had been approved for the treatment and management of Hodgkin’s lymphoma, and monomethylauristatin E had also been studied in several clinical trials based on its ability to form complexes with antibodies and proteins on the membrane. Another analogue—namely, glembatumumabvedotin—has also been recognized for its activity in the management of melanomas, especially those with metastatic breast cancer, due to its association with transmembrane glycoproteins [44]. Polatuzumabvedotin and pinatuzumabvedotin are also being evaluated for their activity in the management of lymphomas and leukemias, due to their direct antibody-directed action on CD 22 and CD 79b proteins (Table 1).

## 4. Marine Drugs in the Management of Diabetes Mellitus

Diabetes mellitus is a chronic metabolic disease that is mainly characterized by blood sugar level elevation and abnormal metabolism of sugar, and it contributes in a major way to mortality and morbidity rates across several developing and developed nations, including India. This disease includes a defect in the functioning of insulin, including its secretion and site of action. The destruction of other microstructures such as neurons, nephrons, and retina depicts the severity of the disease, highlighting its role in affecting nerves, kidney, and eyes. Several cardiovascular disorders and other conditions also occur upon the emergence of diabetes [45]. An increase in reactive oxygen species and oxidative stress is considered as playing a crucial role in the development of diabetes and its associated complications [46]. Food acts as a major source of energy in the form of sugar and helps in maintaining the physiological functioning of the millions of cells in the body. The body regulates sugar by moving it through the cell membrane via two mechanisms: a receptor, which acts as a door, and insulin, which targets the receptors. Type 1 diabetes is a hyperglycemic condition with a defect in insulin, while type 2 diabetes is mainly characterized by increased levels of sugar, mostly due to a defect in receptors [45]. The prevalence of type 2 diabetes is higher globally in comparison with type 1diabetes. It has been estimated that more than 20 million people worldwide have been diagnosed with type 1diabetes, with a predicted annual increase of 2% to 5% every year in several countries. However, type 2 diabetes accounts for approximately 90% to 95% of diabetic cases globally. In the year 2011, over 280 million people were estimated to be affected by type 2 diabetes and the number is assumed to rise to up to 500 million by 2030 [47]. It has become necessary to adopt preventive measures to reduce the burden of this disease on the health and economy of a country. Several synthetic drugs are available in the market to treat this disease, but the medications are not fully effective and are also costly. Moreover, continued use of these drugs can lead to undesirable adverse reactions in the patients. Patients suffering with type 1 diabetes are solely dependent on external insulin injections for their survival and for maintaining a normal life, but it is not comfortable to inject insulin daily. However, type 2 diabetes can be initially managed or controlled by modifications in lifestyle and diet, but type 2 diabetes often requires treatment with oral antidiabetic drugs in the disease’s later stages, and at the end, treatment typically requires insulin injections, which is the most severe scenario. Antioxidants and immune therapy, islet therapy, inhibitors of alpha glucosidase, and other antidiabetic drugs are some of the available therapies to control and manage type 2 diabetes mellitus, but a wide range of side effects also come alongside, which has led to the continued development of novel preventive and regenerative therapies for preventing deficiencies in beta cells mass and for prolong the earlier stage of this disease. Marine sources are being explored due to their promising potential as therapeutic agents in the management of various medical disorders so that they can be employed as a novel or adjuvant therapies. Several marine drugs have been identified in recent years for their exceptional potential for treating or curing diseases. Algae and fish have acted as chief sources of several peptide molecules that possess lipid lowering, anticancer, and anticoagulation properties [48]. In addition, principal antioxidants such as phenolics, carotenoids, and omega 3 fatty acids are also derived from marine seaweeds, crustaceans and fish oil, and bacteria, respectively.

Over 500 marine and freshwater cyanobacteria have been studied for anti-glucosidase and anti-amylase activity, and 38 interesting candidates have also been determined to be fruitful in the management of diabetes [49]. A marine sponge-related bacterium known as *Coralliphaga* has been found to possess major activity in polysaccharide degradation and in the processing of glycolipids and glycoproteins, as it produces a number of glucosidase inhibitors, presenting it as a good target for development in the treatment of diabetes, as well as obesity. Strains of Streptomyces bacteria such as *Streptomyces corchorusii* subspecies rhodomarinus presented fascinating antidiabetic properties by inhibiting the activity of enzyme amylase, while other species of a similar strain led to the production of two novel compounds having N-acetyl-glucosaminidase inhibition properties—namely, Pyrostatins A and B [50]. In addition, to the antidiabetic activity of bacteria, cyanobacteria, and actinomycetes, marine fungi have also been screened to assess whether they might have antidiabetic action. A protein tyrosine phosphatase (PTP1B) inhibitor—namely, aquastatin B—has been obtained from the marine fungus *Cosmospora* species SF-5060, which was isolated from the sediment collected from inter-tides at Gejae Island in Korea [51]. Photosynthetic eukaryotic microalgae, which form a major part of freshwater and marine phytoplankton, have also been confirmed to have significant activity in the management of diabetes mellitus [52,53].

Recent biotechnological advances in aquatic technology have successfully identified promising antidiabetic agents in microalgal species, by virtue of their anti-glycation function. The green microalgal species named *Chlorella* and the diatom *Nitzschia laevis* have been found to have maximum inhibitory effects against the formation of total advanced glycated end products (AGEs)—specifically, N-carboxymethyllysine and pentosidine [54]. The presence of carotenoids such as neoxanthin, antheraxanthin, violaxanthin, and lutein account for the strong AGEs inhibitory activity of Chlorella, while linoleic acid, eicosapentaenoic acid, and arachidonic fatty acids have presented similar bioactivity in *Nitzschia laevis*. The different extracts of these microalgae—mainly *Chlorella zofingiensis*—were tested for their antiglycation activity, and it was determined that the extracts rich in astaxanthin possessed the highest antiglycative and antioxidant activity and hence can be used as a food supplement for the prevention of diabetes in patients [55]. Three strains of microalgae—namely, *Chlorella protothecoides*, *Chlorella zofingiensis*, and the diatom *N. laevis*—have been evaluated to possess protective action against the exogenous and endogenous AGEs in an ARPE-19 cell-based model due to the presence of nutritional ingredients such as carotenoids and omega 3 fatty acids within them. These microalgae also decrease the levels of mRNA expression of vascular endothelial growth factor (VEGF) and matrix metalloproteinase 2 (MMP-2), which are key factors in the etiology of diabetes-induced retinopathy and therefore can be used as a food supplement in the prevention and management of diabetic retinopathy due to presence of carotenoids and omega 3 fatty acids, while also preventing cataract development and further macular degeneration [56].

Corals, sea grasses, fish, shark fusion proteins, sea anemones, salmon skin, and even fish and shellfish wastes have also been evaluated for their antidiabetic properties in the past 15 years. For centuries, macroalgae have been consumed by coastal people as a readily available food, even when its nutritional properties were veiled. In todays’ time, they have been adopted as a healthy lifestyle approach in several countries and are being consumed entirely in the form of extracts or complete foods for their beneficial properties [57]. A number of green, red, and brown algae such as *Palmaria*, *Ecklonia cava*, *Alaria*, *Rhodomela confervoides*, and *Ascophyllum* have presented significant antidiabetic properties. Amylase inhibitory activity was observed in red algae *Palmaria* species phenolic extracts, in combination with antidiabetic properties presented by protein hydrolysates from *Palmaria palmata*. The methanolic extracts of the brown algae *Ecklonia cava*, *Pelvetica siliquosa* also presented diminished levels of glucose in the plasma of the diabetic rats. *Sinularia firma* and *Sinularia erecta*, two soft corals, have also been proven to exert blood glucose lowering activity in diabetic rats exposed to their methanolic extracts, which also prohibited a postprandial rise in hyperglycemia in normal rats [47,58]. Free fatty acids were significantly decreased upon consumption of collagen peptides from marine wild fishes, and they also regulated the nuclear receptors in patients with insulin dependence or type-2 diabetes.

Therefore, it can be concluded that marine products and byproducts, if explored judicially, can be the source of several promising and novel lead molecules in the treatment and management of diabetes (Table 2).

## 5. Marine Natural Products and Cardiovascular Diseases

Cardiovascular disorders (CVDs) are a group of diseases of the blood vessels and heart and include coronary artery disease, atherosclerosis, cerebrovascular disease, hypercholesterolemia, rheumatic heart disease, and several other conditions. They are primarily the leading cause of death worldwide, and 4 out of every 5 deaths related to CVDs are reported to be due to cardiac arrest or stroke. About 17.9 million people die from CVDs every year, which is double the number of deaths due to cancer, and many of these deaths are premature, occurring in patients below 70 years of age. Unhealthy diet, lack of exercise, smoking, alcohol, and other altered lifestyle conditions are the primary causes that initiate the risk of CVDs in the healthy population. The effects may be experienced by patients as increased blood pressure, hypercholesterolemia, obesity, increased glucose in blood, and weight gain. These initial or intermediate risks can be diagnosed in primary healthcare facilities, and if not taken care of or ignored, they can lead to serious conditions of stroke, cardiac arrest, heart failure, and other complications. Increased levels of cholesterol in the body lead to atherosclerosis, which causes a narrowing and clogging of the arteries due to cholesterol and fat deposition, which further leads to coronary artery diseases (CAD). These conditions can also result in hypoxia and myocardial ischemia. The CVDs mainly affect the younger generations and their families in a destructive context and are worthy of more attention to their efficient control and management [59]. The pharmacological interventions used in the management of these diseases include treatment with antiplatelet drugs, statins, or other lipid lowering drugs, anticoagulant drugs, beta blockers, renin angiotensin aldosterone (ACE) inhibitors, diuretics, etc. The use of lipid-lowering agents significantly improves the survival rate in patients affected by coronary artery diseases. The existing therapies that are being used for the prevention of CVDs possess a certain set of limitations and adverse effects and do not provide comprehensive treatment. Hence, owing to a greater population exposed to and affected with these disorders, there comes a dire need to explore and develop other health-friendly treatment approaches that can prevent the disease with minimal side effects. The natural products obtained from marine sources have been considered to be a valuable source for the discovery of new and novel lead molecules by virtue of the diverse chemical structures and pharmacological properties associated with them.

Some of the marine drugs that have been proven to be efficacious in the management of cardiovascular diseases, including coronary artery diseases, are discussed below.

### 5.1. Fucoxanthin

Fucoxanthin is an oxygen-containing carotenoid compound isolated from brown algae. This compound has been studied as a treatment to prevent the over-oxidation of lipids and restrain their accumulation [60]. Fucoxanthin also downregulates transcription factors such as peroxisome proliferator activated receptor (PPAR) and sterol regulatory element-binding protein 1c, which are play a role in adipogenesis. This compound decreases the expression of fatty acid synthase and increases the activity of adipose triglyceride lipase, further leading to the production and phosphorylation of hormones that are sensitive to lipolysis. Furthermore, fucoxanthin also represses the genetic expression of interleukin- 6 and acetyl coenzyme A carboxylase (ACC) and ultimately prohibits the accumulation of adipose tissue, improves resistance to insulin, reduces the thickness of cholesterol, and also affects the concentration of triglycerides in the body. The compound successfully upgraded the functioning of glucose transporter 4 (GLUT 4) and enabled it to consume more energy by oxidizing fatty acids and producing heat. A remarkable fall in the levels of blood glucose was also achieved by its property of enhancing the manifestation of glucokinase (GCK) mRNA and curbing the expression of mRNA phosphoenolpyruvate carboxy kinase. All of these factors help in reducing cardiovascular risks by decreasing lipid concentrations, and fucoxanthin can be used in the management of cardiovascular diseases such as hyperlipidemia, atherosclerosis, coronary artery disease, hypertension, etc. [61]. The illustration of proteins PPAR-α, carnitine, and p-ACC was promoted by fucoxanthin, which led to decreased expression of fatty acid synthase (FAS) on the liver, thereby decreasing the levels of lipids in the bloodstream. Advanced ongoing investigations revealed the beneficial effects of fucoxanthin in preventing lipid increase, and hence controlling and managing cardiovascular diseases.

### 5.2. Saponins

Sea cucumber saponins (SCS) are a glycoside group that comprises of spirostane and triterpene aglycone compounds. They are the secondary metabolites that are obtained from the sea and have been demonstrated to possess anti-atherosclerotic activity. They mainly act by regulating the metabolism of lipids and glucose in the body. The main functional constituent of sea cucumber saponins is adiponectin, which is believed to restore lipid and glucose metabolism by the promotion of sirtuin 1 (SIRT1), which further restricts the activity of sterol regulatory element-binding protein (SREBP)-1c and FAS. All of these factors ultimately promote hepatic fatty acid oxidation. The mRNA levels of GCK can be stimulated by SIRT 1 in the liver, which results in the catalysis of phosphorylation of glucose and is the first move towards glycolysis [62]. To conclude, the SCSs ameliorate the metabolism of lipids and glucose by altering the activity of SIRT 1, FAS, GCK, PPAR-α upon secretion of adiponectin and hence suppressing lipid production and synthesis and enhancing oxidation of fatty acids, thereby reducing the risk of cardiovascular diseases.

### 5.3. Astaxanthin

Astaxanthin, also classified as xanthophyll, is a naturally occurring microalgae and marine compound renowned for its bioactivity. The unique structure of the astaxanthin molecule accounts for its characteristically strong antioxidant activity and enables it to quench oxygen in singlet states and release free radicals. The molecule depicts its beneficial role in several biological activities such as amelioration of oxidative stress, decrease in inflammation, and altered lipid and glucose concentrations. Astaxanthin induces the oxidation of high-density lipoproteins (HDL) and adiponectin levels and suppresses the oxidation of low-density lipoproteins (LDL), i.e., promotes good cholesterol. The molecule possesses atherosclerosis-protective effects, as it can lighten the proportion of plaque formation and cholesterol in the aortic sinus region and aorta in mice, respectively. The levels of thiobarbituric acid (TBARS) were also decreased owing to the antioxidant activity of the compound, and hence it acts as a strong oxidation resistance substance [63]. The macrophage activation was suppressed, while granulocytosis of neutrophils was promoted by astaxanthin to induce phagocytosis and sterilization. Hence, this compound is inferred as being able to sustain less damage in body and to control the levels of lipid upon its elevation.

### 5.4. Xyloketal B

Xyloketal Bis a new style of an extraordinary structure containing marine compound isolated from the *Xylaria* species. This compound prevents endotheliocytes from suffering oxidative injury by inducing the oxidation of low-density lipoprotein due to suppression of NADPH oxidase-derived reactive oxygen species synthesis, and it facilitates the further release of nitric oxide. The endothelial dysfunction due to oxidative stress is also controlled. The compound has been determined to be effective in suppressing the levels of oxidative stress in vascular tissues, to improve the integrity of endothelial cells that were previously injured due to conditions of stress, and to promote vaso relaxation. Xyloketal B also reduced the levels of lipids by promoting their oxidation and by suppressing their accumulation [64].

The consumption and demand of marine products has increased gradually with development in the recent years. Marine products are believed to contain more bioactive substances, as they have a tendency to survive in the complex environment of the ocean. Extensive research in products obtained from marine resources has confirmed the role of marine drugs in the management of cardiovascular diseases, which has also encouraged researchers to hunt for and discover more safe and novel lead molecules (Table 3).

## 6. Marine Drugs in Neurodegeneration

**Figure 2:**
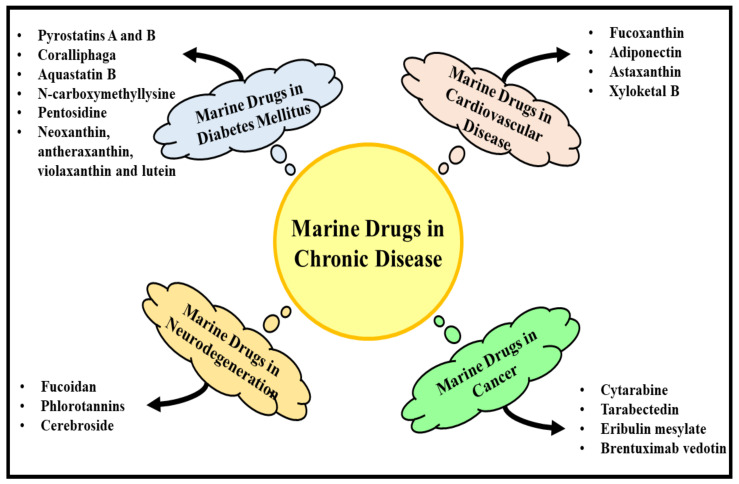
This figure provides a descriptive overview of the marine drugs used in diabetes mellitus, cancer, neurodegenerative disease, and cardiovascular disorders.

Neurodegenerative diseases are the greatest medical challenge faced by researchers in the 21st century. They are a group of heterogeneous late onset disorders that are caused by progressive dysfunction of neuronal cells and their death, leading to cognitive impairment and altered movement. With increasing age, the incidence of neurodegenerative diseases also increases, and they are more common in the elderly age group of patients. The main factor that is associated with the process of neurodegeneration is aging [65,66]. These diseases are mainly characterized by loss in the functioning of neurons and the formation of misfolded proteins, accompanied by their aggregation, extracellular intracellular deposits, and neuronal cell death. Excessive release of reactive oxygen species (ROS) and neuroinflammation contribute chiefly to the pathophysiology of different neurodegenerative diseases and depict a direct consequence of perturbation in the homeostasis of the central nervous system (CNS) [67]. Effective treatments are being explored, and efforts are being made to manage age-related degeneration and promote stability in patients. The current therapies employed in the management of neurodegenerative disorders improve quality of life to only a certain extent. Hence, the field calls for research and development that explores and discovers new and safer lead molecules with novel mechanisms of action by targeting certain physiological pathways and improving the condition of the patients. As the interest rises in natural products, the attention also drifts towards the sea, as it has the greatest diversity of plants and animals and is the least explored. Hence, below are described some of the promising compounds obtained from the sea and a description of their consumption as supplemental therapies for a role in the management and prevention of neurodegenerative diseases [68,69] (Figure 2).

The venom of sea snail *Conus Magus* led to the derivation of a synthetic form of the peptideziconotide, which is a selective N-type calcium channel blocker and is used in conditions of chronic pain owing to its antinociceptive effect without the development of tolerance, as is witnessed in other opioids and morphine. This fact represents a significant advantage of this marine-derived product for use in the long-term management of conditions of pain. Many other drugs are also being identified from the ocean and extracts of marine invertebrates. Several compounds isolated from marine sources have been studied and found to possess modulatory action on the CNS, and can thus be involved in the management of neurodegenerative conditions. For instance, potent cholinesterase inhibitors were isolated from sponges, mollusks, algae, bryozoans, cnidarians echinoderms, and tunicates. Xestospongia, xestosaprols, Indonesian marine sponges, tasiamide B cyanobacteria, carteriospongia-derived carteriosulfonic acids, petrosamine sponges, *Reniera sarai* sponges, octocorals, and lophotoxin are some of the marine-derived bioactive products that are assumed to be hypothetical candidates for development as novel drugs for themanagement of CNS disorders. Marine source-derived cholinesterase inhibitors can be used in the management of mild and moderate atopic dermatitis; however, their use can only reduce symptoms in a patient and cannot arrest the progression of the disease [70,71]. Another marine compound named conotoxins is an antagonist of CNS receptors and exhibits a significant role in various physiological processes such as memory, learning, and attention. Some of the marine drugs used prominently in the management of neurodegenerative disease are listed below.

### 6.1. Fucoidan

Fucoidan is a polysaccharide isolated from brown algae—namely, *Saccharina japonica*—and comprises sugar, fucose, and sulfate content [72]. This compound exhibits a protective effect in the management of Parkinson’s disease due to its interaction with 1-methyl-4-phenyl-1,2,3,6- tetrahydropyridine (MPTP), and it thereby effectively improves motor impairment. The compound also successfully was able to counteract the depletion of dopaminergic neurons in the striatal region. Upon treatment with this compound, the morphology of neuronal cells was preserved and mitochondrial activity was found to be increased. The pharmacological mechanism of this protective action is unclear; however, it is demonstrated to be due to its antioxidant activity, as it prevents the generation of reactive oxygen species and other mechanisms such as anti-inflammation [73].

### 6.2. Seaweeds

Seaweed is another marine compound that is believed to have exceptional bioactive properties due to its antioxidant nature. The different extracts of seaweeds were tested, and their neuroprotective effect was elucidated as being owed to their anti-apoptotic mechanism. The extracts substantially prevented dopaminergic neurotoxicity, and an increase in cell viability was noted, leading to its use in the management of Parkinson’s disease. Phlorotannins, exclusively found in brown seaweeds, are responsible for the antioxidant activity of this compound. *Codium Tomentosum* has emerged as a promising neuroprotective seaweed that has anti-genotoxic and anti-oxidative properties. The compound readily scavenges both nitrogen and oxygen free radical species and contains different organic acids, such as malic acid, aconitic acid, oxalic acid, malonic and fumaric acids, phenolic compounds, and secondary metabolites [74,75].

### 6.3. Cerebrosides

Cerebrosides are a category of neutral glycosphingolipids that are mainly present in marine species. These compounds are extensively found in the brain and convert into ceramides, which are further transitioned into sphingomyelins, sulfatides, and other associated glycosphingolipids. These compounds are highly crucial in maintaining functioning and homeostasis in the brain. The unique structure of this compound is composed of three units—a polar monosaccharide head, long chain of sphingoid base, and amide-linked fatty acids—that contribute to the biological activities of this compound that has attracted the attention of the pharmaceutical industry. Sea cucumber cerebrosides are believed to improve cognitive functioning and synaptic plasticity and to attenuate hyperphosphorylation of tau proteins by regulating the neurotrophic pathway. Administering sea cucumber cerebrosides as a food supplement may present neuroprotective effects and may even improve cognitive functioning. The compound has also been reported to have activity useful in the management of Alzheimer’s and Parkinson’s diseases. Certain marine compounds can be further synthesized through this molecule to produce more specific biological effects [76,77,78] (Table 4 and Table 5), Figure 2.

## 7. Future Prospects of Marine Drugs and Drug Candidates Obtained from the Ocean

For decades, a strong effort has been undertaken by the pharmaceutical industry and academia to derive therapeutically active marine drugs [78]. Eleven drugs with their origin in marine natural sources have successfully navigated their way through development and into the market, among which at least five can be used in the management of cancer, such as cytarabine (Cytosar-U), eribulin mesylate (Halaven), and trabectedin (ET-473, Yondelis) and including antibody drug conjugates such as polatuzumabvedotin (Polivy) and brentuximab vedotin (Adcetris). Other drugs such as Epanova, Lovaza, and Vascepa have also been used in treatment of hypertriglyceridemia, while vidarabine (Vira-A) and iota-carageenan (Carragelose) have been used as antiviral drugs and zinconotide (Prialt) has been used for ameliorating chronic pain. More than 20 drug candidates have been obtained from marine natural products as of now and which are under investigation in different phases of clinical trials, including several drug leads that are being studied extensively for their activity in pre-clinical studies [79,80,81,82]. The link between a direct therapeutic target (also called the binding partner) and a mechanism of bioactive action associated with the disease serves as an incentive for conducting preclinical and clinical studies. The use of novel techniques in the identification of targets and the capture of associated mechanisms of action act as a game changer and can lead to the implementation of early stages of discovery efforts for new drug leads [83,84]. Marine natural products are highly diverse and thus present a greater opportunity for discovering a wide number of first-in-class therapeutic agents that comprise proteins and other unusual biological targets, such as lipid membranes, sterols, etc. [85]. These targets further lead to the production of the effective antibody drug conjugates that are commonly used in the management of several chronic diseases [86]. The marine environment offers a rich arsenal of bioweapons that possess diverse structure and functional properties and that are under investigation in different stages of clinical trials. Aplidin is a first-in-class cyclic depsipeptideplitidepsin isolated from marine tunicate named *Aplidium albicans,* and it interacts strongly with eukaryotic elongation factor 1A2 (eEF1A2) in the cells of a tumor [87]. This marine drug is being studied in combination with dexamethasone for its efficacy in the management and treatment of relapsed and refractory multiple myeloma and has successfully reached phase III clinical trials [88].

Tectin or tetrodotoxin is a derivative of guanidine and comprises a skeleton of highly oxygenated carbon obtained from puffer fish, which produce one of the most popular marine toxins [89]. It is mainly isolated from the liver, skin, and ovaries of puffer fish belonging to family *Tetraodontidae*. The toxin accumulates in the body through the food chain in marine oceans and is derived from specific endosymbiotic bacteria, mainly pseudomonas and vibrio species. Tetrodotoxin acts by competitively and selectively hindering the activity of voltage-gated sodium channels (VGSCs) in the cell membrane of the nerve cells, which prevents propagation of action potential due to depolarization and causes loss of sensation [90]. The toxin is being used to an extensive degree due to its chemical nature in characterizing voltage-gated sodium channels, and it is also applied as an analgesic to cure pain. Tetrodotoxin successfully completed two phase III safety and efficacy studies in 2012 for its use in the management of moderate-to-severe inadequately controlled pain related to cancer, the results of which are still awaited [91].

Another marine drug that is commonly used for its therapeutic use is plinabulin, a synthetic analogue of a naturally occurring aromatic alkaloid—namely, halimide—mainly obtained from a marine fungus belonging to a species of *Aspergillus* [92,93]. It is mainly collected from the waters of the Philippine islands. Plinabulin targets tubulin monomer near the colchicine binding site and inhibits polymerization of tubulin, which further causes tumor vascular network destabilization and reduces blood flow to the tumor region, thereby preventing tumor progression [94]. The marine drug is being investigated in phase III clinical trials for its apoptotic effects in tumor cells and vascular disruption activity in the management and treatment of non-small cell lung cancer [84,95].

**Figure 3:**
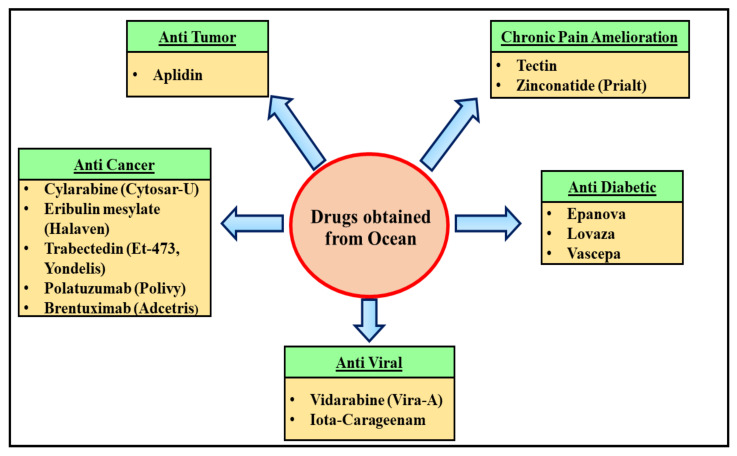
Marine drugs obtained from the ocean and their therapeutic uses in the management of conditions, starting from pain to life threatening diseases such as cancer.

The marine bacteria named *Salinispora arenicola* and *Salinispora tropica* led to isolation of a potent proteasome inhibitor, Salinosporamide A or Marizomib. Proteasomes received a huge amount of attention after the success of the first proteasome inhibitor, the drug bortezomib, which was approved for the management of cancer and has been developed as a class of proteins in anticancer therapy [96]. In the year 2012, an analog of epoxomicin—namely, carfilzomib (Kyprolis)—was approved as an anticancer drug [97,98]. Carfilzomib was isolated from strains of actinomyces and acted as a potent proteasome inhibitor, suggesting its eminent role as a marine-derived therapeutic agent. Within 3 years of its discovery, Salinosporamide A successfully entered phase I clinical trials for investigation of its activity in the management of multiple myeloma, and it was further transitioned into phase 2 trials that have also been completed (Figure 3).

## 8. Conclusions

The structure–activity relationship (SAR) of the isolated marine natural products and their derivatives revealed a scaffold that increased the activity of the chemical compounds obtained for the therapeutic management of several disorders. The marine products obtained can also be optimized further in laboratories to synthesize more precise and potent lead molecules that can be effectively employed for their disease targeting activity and also to fight against pathogens. Advances in techniques have led to an increased understanding and broadening of the biological activities of these drugs in the management of chronic diseases, including cancers. The interest of researchers in marine products is flourishing, which has led to the budding of innovative solutions for overcoming the challenges of their extraction and isolation. Marine drugs exert significant therapeutic effects such as antioxidant, anti-inflammatory, anti-apoptotic, antimicrobial, antidiabetic, and antihyperlipidemic properties. The consequences of administering these compounds are extremely less severe when considered in the context of the intensity of a disease and other alternative therapies.

## Figures and Tables

**Table 1:** The below table lists the marine drugs used in cancer treatment and their respective structures.

Name	Structure
Cytarabine	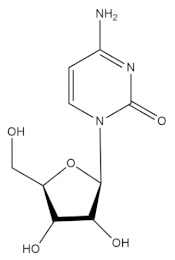
Trabectedin	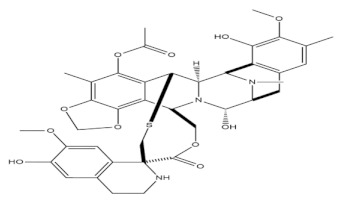
Eribulin Mesylate	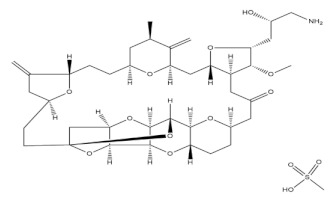
Brentuximab vedotin	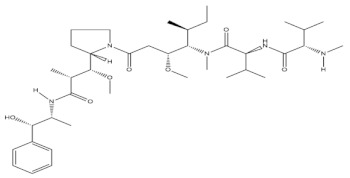

**Table 2:** The below table lists the marine drugs used in diabetes mellitus and their respective structures.

Name	Structure
Pyrostatins A and B	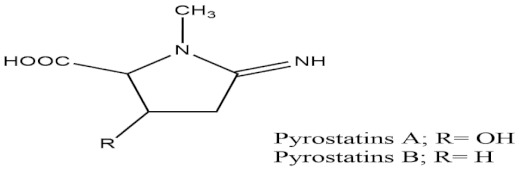
N-carboxymethyllysine	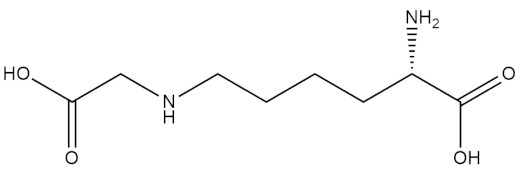
Pentosidine	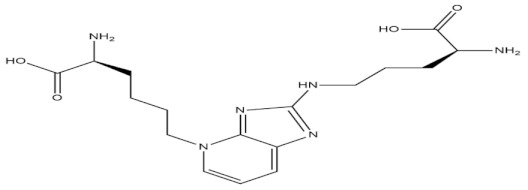
neoxanthin	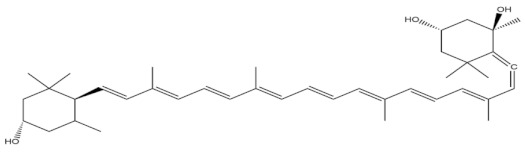
antheraxanthin	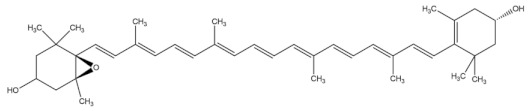
violaxanthin	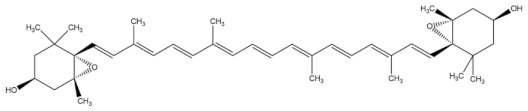
Lutein	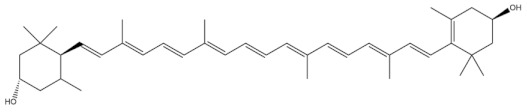

**Table 3:** The below table lists the marine drugs used in cardiovascular disorders with their respective structures.

Name	Structure
Fucoxanthin	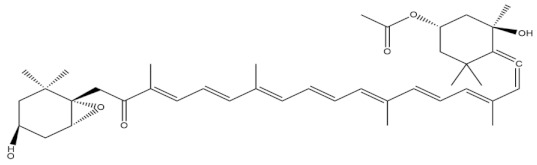
adiponectin	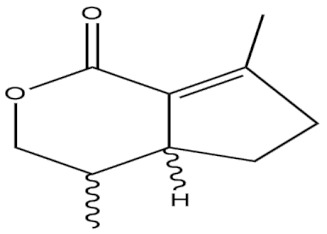
Astaxanthin	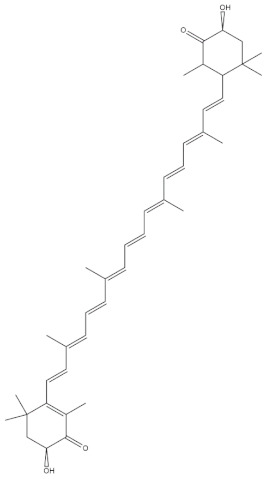
Xyloketal B	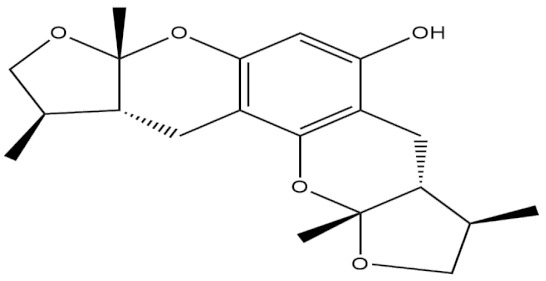

**Table 4:** The below table lists the marine drugs used in neurodegenerative diseases with their respective structures.

Name	Structure
Fucoidan	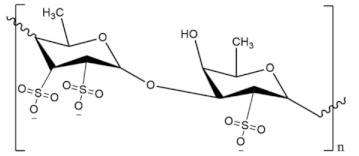
Phlorotannins	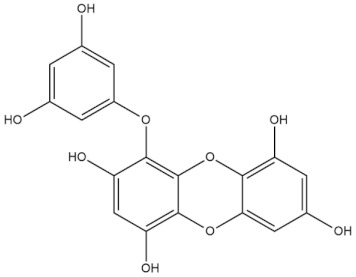
Cerebrosides	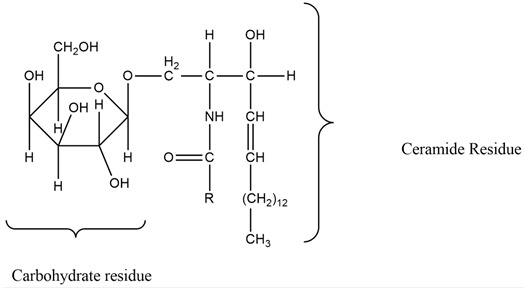

**Table 5:** The below table lists compounds obtained from marine sources and their uses.

Name of the Compound	Source	Scientific Name	Uses	References
Ziconotide	Cone snails	*Conidae*	Management of spinal cord injury-mediated chronic pain	[4]
Hemiasterlin, discodermolide	Marine sponges	*Spongia officinalis*	Anticancer, anti-inflammatory, antibiotic	[11]
Cytarabine	Caribbean sponge	*Cryptotethya crypta*	Anticancer	[32,33,34]
Trabectedin	Caribbean ascidian	*Ecteinascidia turbinate*	Anticancer	[35]
Eribulin Mesylate	Sponge	*Halichondria okadai*	Anticancer	[36]
Brentuximab vedotin	Gastropod mollusk	*Dolabella auriculria*	Anticancer	[36,37]
	marine sponge bacterium	*Coralliphaga*	Antidiabetic	[43]
Aquastatin B	Marine fungi	*Cosmospora* species SF-5060	Antidiabetic	[44]
	Chlorella and diatom	*Nitzschia laevis*	Antidiabetic	[47]
Astaxanthin		*Chlorella zofingiensis*	Antidiabetic	[48]
	Green, red and brown algae	*Palmaria*, *Ecklonia cava*, *Alaria*, *Rhodomela confervoides* and *Ascophyllum*	Antidiabetic	[40,51]
Fucoxanthin	Brown algae	*Phaeophyceae*	Antihyperlipidemic	[53]
Spirostaneand triterpene aglycone compounds	Sea cucumber saponins	*Holothurialessoni*	Anti-atherosclerotic	[55]
Xyloketal B		*Xylaria* species	Antioxidant, antihyperlipidemic	[57]
Fucoidan	Brown algae	*Saccharina japonica*	Parkinson disease, anti-inflammation	[63]
	Seaweed	*Codium Tomentosum*	Neuroprotective, anti-apoptotic	[64,65]
Aplidin	Marine tunicate	*Aplidium albicans*	Anticancer	[76]
Tetrodotoxin	Puffer fish	*Tetraodontidae*	Analgesic	[79,80]
Plinabulin	Marine fungus	belonging to species of *Aspergillus*	Under investigation in clinical trials phase III as antitumor	[81,82,83,84]
Salinosporamide A or Marizomib	Marine bacteria	*Salinispora arenicola* and *Salinispora tropica*	Proteasome inhibitor	[85,86,87]

## Data Availability

Not applicable.
